# Endophytic Fungal Community of Tobacco Leaves and Their Potential Role in the Formation of “Cherry-Red” Tobacco

**DOI:** 10.3389/fmicb.2021.658116

**Published:** 2021-07-16

**Authors:** Yonglei Jiang, Xing Chen, Gaokun Zhao, Jiahong Liu, Yan Xie, Yong Li, Huaguo Gu, Congming Zou

**Affiliations:** ^1^Yunnan Academy of Tobacco Agricultural Sciences, Kunming, China; ^2^Qujing Branch, Yunnan Tobacco Company, Qujing, China

**Keywords:** “cherry-red” tobacco, endophytic fungi, community diversity, taxonomic biomarker, co-occurrence network

## Abstract

“Cherry-red” tobacco is the superior variant of tobacco, appearing with the apperance of red dapples on cured leaves due to the demethylation of nicotine to nornicotine during maturation and curing. Fungi are known to have the capacity to convert nicotine to nornicotine. However, an endophytic fungal community of “cherry-red” tobacco has never been reported to our best knowledge. Here, we sampled mature leaves from both “cherry-red” and ordinary tobacco at lower, center, and upper plant sections, and we analyzed the ITS diversity using high-throughput sequencing. Results revealed a significantly different fungal community of foliar endophyte in “cherry-red” and ordinary tobacco. In comparison to the ordinary control, higher diversity and a co-occurrence network complex were found in “cherry-red” samples, especially in the center and upper leaves, where the red dapples mainly emerged. More taxa were enriched in the “cherry-red” than ordinary tobacco leaves at all plant sections. In particular, *Aspergillus*, some strains of which are reported capable of converting nicotine to nornicotine, was specifically enriched in upper “cherry-red” tobacco leaves, which showed most red dapples after curing. A less robust network structure was detected in the “cherry-red” tobacco compared to ordinary tobacco. The nearest taxon index (NTI) and β NTI indicated that the local community structuration of tobacco endophytic fungi mainly driven by deterministic process, while the community turnover among plant sections was stochastic. In conclusion, our study provides the earliest information of endophytic fungal community in “cherry-red” tobacco leaf, and the community diversity, composition, and network features are synchronously varied with the appearance of red dapples, which is suggestive of their relationship to the formation of “cherry-red” tobacco.

## Introduction

Endophytes are microorganisms that live freely in the cells or intercellular spaces of plants without harming the host (Rodriguez et al., 2009; Reinhold-Hurek and Hurek, 2011). These bacteria and fungi can significantly shape the metabolism, fitness, and evolution of plants as well their interaction with other organisms, and they are thus critical to plant physiology (Omacini et al., 2001; Rodriguez et al., 2009; Nuangmek et al., 2021). In agriculture ecosystems, fungal endophytes increase the fertilizer use efficiency of crops and improve the resistance to environmental stresses and diseases (Rodriguez et al., 2009; Calvo et al., 2014; Zavala-Gonzalez et al., 2017; Zhou et al., 2021). In recent years, as conventional chemical fertilizer and pesticides meet their bottleneck in both economic and environmental benefits, exploring to the maximum the functions of the plant microbiome, including the endophytes, is urgent for sustainable and efficient agroecosystems (Toju et al., 2018).

The endophytic community of a certain crop type is affected by the varieties (Zhang et al., 2019; Mina et al., 2020). For example, Zhang et al. (2019) found a large population of bacteria was specifically enriched in the root of *indica* rather than the *japonica* varieties, which is proved to account for the high nitrogen use efficiency of *indica* rice. Beyond the variety, microbial communities even varied among compartments of individual plants, i.e., root, leaf, and stem, etc., as they represent distinct ecological niches (Novas et al., 2011; Xiong et al., 2021). Since the variations in the endophytic community are related to nutrient content and secondary metabolites (Ludwig-Muller, 2015; Marques et al., 2015), they are suggested to play a role in determining the production quality of agricultural crops (Rodriguez et al., 2009; Wang et al., 2011; Rho et al., 2020; Taghinasab and Jabaji, 2020). Despite the function of endophytes in the growth and metabolism of plant host remains largely postulated until now, investigation of the endophytic community is valuable and intriguing, especially considering that the relationship between production quality and endophytes are hereditarily repeatable in many cases (Wang et al., 2011; Droby and Wisniewski, 2018). However, the existence and functions of endophytes in harvested commodities remand limited studied (Droby and Wisniewski, 2018).

Tobacco is one of the most important industrial crops. “Cherry red” tobacco is a superior variant that shows red dapples in the cured leaves due to the enrichment of nornicotine (Hall et al., 1965). In most commercial tobacco, nicotine makes up 90–95% of the total alkaloids (Armstrong et al., 1998). However, in “cherry-red” tobacco, most of the nicotine is demethylated. As reported by Liu et al. (2020), nicotine accounted for only 6.4% of all the aroma in nornicotine-enriched tobacco compared to 46.5% in ordinary tobacco. Low nicotine content makes the “cherry-red” tobacco taste mellow, being less fragrant and producing less irritation, and it thus has wide appeal for Chinese consumers (Lin et al., 2016; China Cigarette Website, 2018). The producing ratio of “cherry-red” leaves varied among plant sections. Generally, most red dapples are found in the upper leaves after being cured, while they are found less in the lower leaves. Mechanisms underlying the generation of “cherry-red” tobacco have remained unrevealed until now. Nevertheless, endophytic fungi have been reported to play a role in the conversion of nicotine to nornicotine (Uchida et al., 1983). However, until now, we know nothing about the endophytic fungal community of “cherry-red” tobacco.

To address this problem, we collected ordinary and “cherry-red” tobacco leaves from the same field. High-throughput sequencing was used to analyze the fungal community of the endophytes. The taxonomic biomarkers of “cherry-red” tobacco were recognized using linear discriminant analysis effect size (LEfSe). The co-occurrence network and community structuring process were analyzed to figure the community assembly mechanisms.

## Materials and Methods

### Experimental Design

**FIGURE 1:**
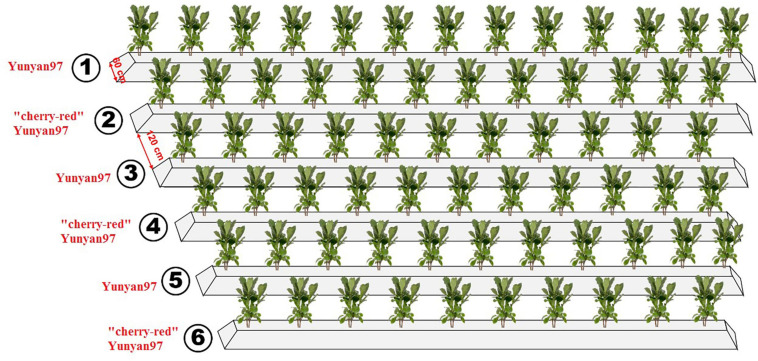
Conceptual graph showing the layout of plots cultivating Yunyan97 and “cherry-red” Yunyan97 fields in the field.

The experimental field was allocated at the Yanhe Research Farm of Yunnan Tobacco Agronomy Research Center, Yunan Provence, China (24°14′N, 102°30′E). For the study, 50-day-old seedlings of Yunyan97 and “cherry-red” Yunyan97 were transplanted to six parallel ridges in May 2020, with Yunyan97 (ordinary, CK) in ridge one, three, and five and “cherry-red” Yunyan97 (“cherry-red”, ZS) in ridge two, four, and six (Figure 1). The ridges had a bottom width of 60 cm and intervals of 120 cm between each other. In total, 154 seedlings were planted in each ridge. The field was fertilized by 105 kg N ha^–1^ and the N:P:K ratio of 1:1.5:3. The fertilization was divided into two times: 10.8 g KNO_3_ to each plant after 30 days of transplanting and all remaining fertilizers applied during the transplanting. All the tobacco plants were exposed to the same management until harvest. To ensure the nutrient supply, 20–22 effective leaves remained for each plant.

### Leaf Sampling

Sampling of ordinary and “cherry-red” tobacco leaves was performed 5 days after the pinching. For each ridge, three plants were randomly chosen as subsamples, which were combined into one sample in the following. The lower (counted from bottom to top: 3rd–4th leaves, L), center (10th–12th leaves, C), and upper (19th–20th leaves, U) leaves were cut off using sterilized surgical scissors at the petiole. Part of the leaves from different plants and different sections were separately packed using aluminum foil, immediately transport to the laboratory in the liquid nitrogen, and stored at −80°C for DNA extraction. The remands were used for baking to confirm the presence of the red dapples of “cherry-red” tobacco (Results shown in Supplementary Figure 1).

### DNA Extraction, Amplicon, and High-Throughput Sequencing

The leaves were surface sterilized using 2% sodium hypochlorite for 10 min, flowed for 2 min in 75% ethanol, and then triply washed using sterilized water. After being cut into pieces of about 1 cm, 100 mg of the three subsamples from the same ridge were combined into one sample, and we then made three replicates for each plant section of CK and ZS. All samples were well ground in liquid nitrogen, and 100 mg of each sample was transported to a 2 ml centrifuge tube. Total DNA was extracted using the Qiagen PowerSoil DNA kit following the manufacturer’s certificate. The DNA concentration and quality were checked on 2% agarose gel.

The ITS1 of fungi was amplified using the primers of ITS1-F (CTTGGTCATTTAGAGGAAGTAA) and ITS2aR (GCTGCGTTCTTCATCGATGC; Al-Sadi and Kazerooni, 2018). The PCR mixture contained 1 μl of DNA template (20 ng μl^–1^), 10 μl 2 × Phusion^®^ High-Fidelity PCR Master Mix (Biolabs, New England), 0.15 μl forward and reverse primers, and 8.7 μl sterilized ddH_2_O. For each sample, a unique barcode was added to the primers at the 5’ end. The PCR condition was as follows: 98°C for 1 min, followed by 30 cycles at 98°C for 10 s, 50°C for 30 s, and 72°C for 30 s, and a final extension at 72°C for 5 min. Sequencing libraries were prepared using the gel-purified PCR production and sequenced using the Illunima NovaSeq platform using the pair ends method (250 bp) by Novogene Co., Ltd. (Beijing, China).

### Reads Processing and Analyses

Paired-end reads from the original DNA fragments are merged by using FLASH (Magoč and Salzberg, 2011) and assigned to each sample according to the unique barcodes. Sequences were then transported to QIIME for further processing (Caporaso et al., 2010). First, reads were filtered to remove the low-quality sequences (Length < 150, Q < 30) and chimeras. Then, *de novo* OTUs were pick at the similarity cutoff of 97%. We pick a representative sequence for each OTU and use the RDP classifier to annotate taxonomic information for each representative sequence (Wang et al., 2007). Before further analysis, singletons and reads affiliated with the host genome were removed from the OTU table. The raw sequences in this study have been deposited in the Sequence Read Archive of NCBI under the accession number PRJNA698403.

Data analyses were performed using R 4.0.3^1^. The sequencing depth was rarefied to 41679 reads per sample using the *rrarefy* function in the *vegan* package. The α diversity, including observed OTUs, Chao1, and Shannon indices, was calculated by the *estimate_richness* function in *phyloseq* using the default parameters. Non-metric multidimensional scaling analysis (NMDS) was performed using *metaMDS* function in *vegan* based on the Bray-Curtis distance. The community data were auto transformed using the built-in standardizing protocol in the function, and the minimum number of random starts in search of the stable solution was set to 20. To construct the co-occurrence network, we used Spearman’s correlation between the relative abundance of each OTU using the *rcorr* function in *Hmisc*. A cutoff of 0.72 was chosen based on the random matrix theory using *rm.get.threshold* function in *RMThreshold* based on the significant relationships (*p* < 0.05; Luo et al., 2006). A global network was constructed with all samples using the *graph_from_adjacADcy_matrix* function in *igraph*. Subnetwork of each sample was extracted from the global network based on the vertex. The network features were calculated using the *igraph* package. Gephi 0.9.2^2^ was used to visualize the networks. LEfSe and community structuring mechanism analyses were performed using the Galaxy pipeline^3^. Taxa with LDA scores higher than two were identified as biomarkers in LEfSe. To test the significant level of the effect of tobacco type (ordinary and “cherry-red”) and plant section on the α diversity indices, network features, and indices of community structuration, Wilcoxon was performed using the *wilcox.test* function. Meanwhile, for NMDS, the significant difference between treatments was tested by PERMANOVA using the *adonis* in the *vegan* package with 999 times random permutations.

## Results

### Diversity and Taxonomic Composition

**FIGURE 2:**
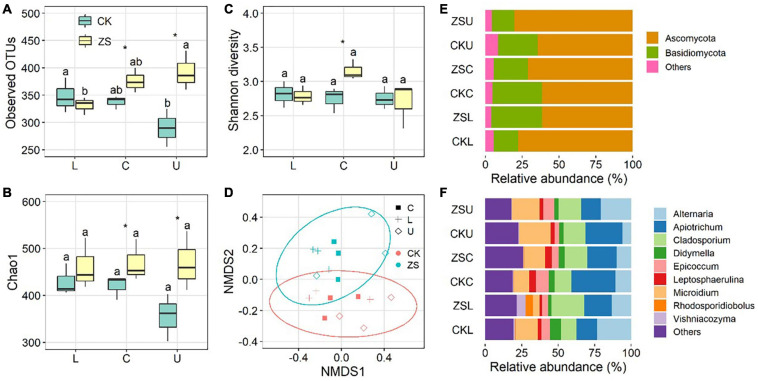
Observed OTUs **(A)**, Chao1 index **(B)**, Shannon diversity **(C)**, Non-metric multidimensional scaling analysis, **(D)** and the relative abundance at phylum **(E)** and genus **(F)** level of endophytic fungal community in ordinary (CK) and nornicotine-enriched (ZS) tobacco leaves at lower (L), center (C), and upper (U) plant sections. Different letters above the boxes indicated significant differences among plant sections from the same tobacco types (CK or ZS), while the significant differences between CK and ZS at the same plant sections are marked by asterisks (*p* < 0.05).

The fungal endophytic richness in CK decreased from the lower to upper plant section, as indicated by both observed OTUs (Figure 2A) and the Chao1 index (Figure 2B). A reverse trend was detected for ZS, i.e., highest at the upper section and lowest at the lower section (Figures 2A,B). However, a significant difference was only found for observed OTUs (*p* < 0.05). The fungal richness was similar in CK and ZS at the lower section, whereas it was significantly higher in ZS than CK at the center and upper sections (*p* < 0.05). Tobacco type and leaf section had a smaller influence on Shannon diversity than OTU richness, showing no significant difference between all samples except for a higher value in “cherry-red” than ordinary tobacco at the center section. NMDS based on Bray–Curtis dissimilarities combining with PERMANOVA revealed a significant difference of the fungal community between ordinary and “cherry-red” tobacco leaf endophytes (*p* < 0.05, Figure 2D).

At the phylum level, *Ascomycota* (61.4–80.5%) and *Basidiomycota* (15.2–33.9%) dominated the endophytic fungi of both ordinary and “cherry-red” tobacco. Higher relative abundances of *Ascomycota* and lower *Basidiomycota* were observed in ZS than CK at the lower section (Figure 2E). However, at the center and upper sections, the relative abundance of *Ascomycota* was higher in CK than ZS. *Altermaria*, *Apiotrichum*, *Cladosporium*, and *Microdium* were the most abundant genera in both tobacco types (Figure 2F). Among the dominant genera, *Apiotrichum* was significantly lower in CK than ZS at the lower section, whereas this was reversed at the center and upper sections (*p* < 0.05).

### Taxonomic Biomarkers of Ordinary and “Cherry-Red” Tobacco Leaves

**FIGURE 3:**
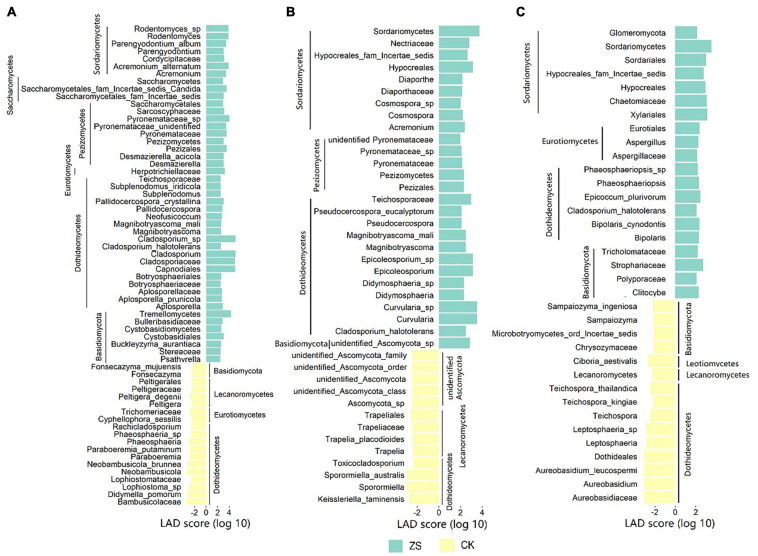
Taxonomic biomarker of the endophytic fungal community in ordinary (CK) and nornicotine-enriched (ZS) tobacco leaves at lower **(A)**, center **(B)**, and upper **(C)** plant sections revealed by linear discriminant analysis effect size (LEfSe).

At the threshold of 2, LEfSe analysis revealed 64 (4 classes, 6 orders, 15 families, 19 genera, and 20 species), 40 (3 classes, 5 orders, 7 families, 13 genera, and 12 species), and 35 (1 phylum, 2 classes, 6 orders, 8 families, 8 genera, and 10 species) statistically significant fungal clades in the lower, center and upper leaves, respectively (Supplementary Figure 2). The taxa enriched in CK or ZS were taken as the taxonomic biomarkers for each other. As shown in Figure 3 and Supplementary Figure 2, more taxonomic biomarkers were detected in ZS than CK, which was 45 *vs* 19 in the lower, 27 *vs* 13 in the center, and 20 *vs* 15 in the upper leaves. Taxa belonging to *Dothideomycetes* were important biomarkers in all samples. Another biomarker-emerging class shared by CK and ZS was *Basidiomycota*, which more frequently appeared in ZS than CK. The *Lecanoromycetes* taxa were distinguished at all the sections of CK but never recognized in ZS samples. Whilst, *Sordariomycetes* biomarkers were only shared by ZS samples from all sections. Moreover, there were three biomarkers (*Hypocreales*, *Hypocreales*_fam_Incertae_sedis, and *Sordariomycetes*) specifically shared by the fungal community in the center and upper leaves of ZS, all belonging to *Sordariomycetes*. We found no unique fungal biomarker in the center leaf of ZS, but *Asperigilaceae* biomarkers (*Aspergillus, Eurotiales*, and *Asperigilaceae*) were specifically found in ZS at the upper section, which showed the most red dapples after cured (Supplementary Figure 1).

### Co-occurrence Network

**FIGURE 4:**
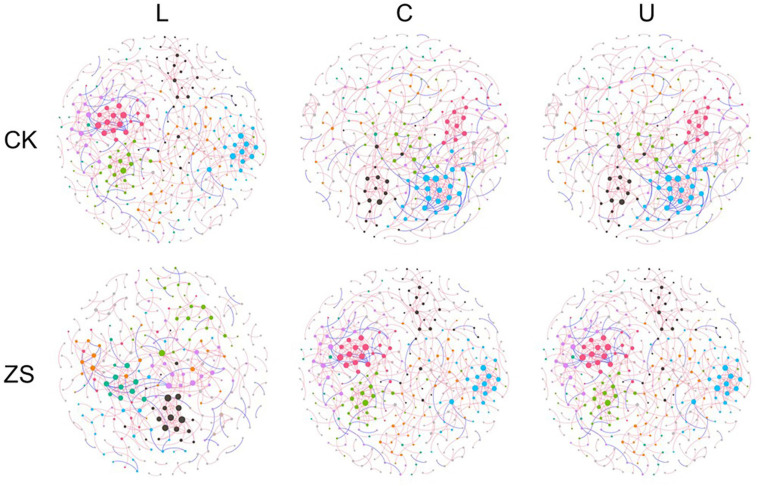
Networks of the endophytic fungal community in ordinary (CK) and nornicotine-enriched (ZS) tobacco leaves at lower (L), center (C), and upper (U) plant sections. Positive and negative links are colored red and blue, respectively. Nodes clustered to the same module in a network are indicated by the same color; however, the colors among networks are not comparable.

**FIGURE 5:**
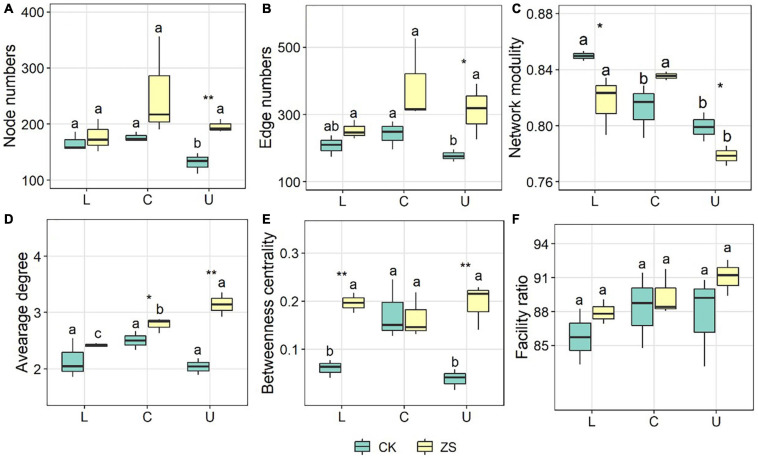
Network features of the endophytic fungal community in ordinary (CK) and nornicotine-enriched (ZS) tobacco leaves at lower (L), center (C), and upper(U) plant sections. Different letters above the boxes indicated significant differences among plant sections from CK or ZS tobacco, while the significant differencesbetween CK and ZS are marked by asterisks (*p* < 0.05). **(A)** node number; **(B)** edge number; **(C)** network modularity; **(D)** average degree; **(E)** betweenness centrality; **(F)** facility ratio.

The co-occurrence network of ZS samples had more nodes, more edges, and a higher average degree in comparison to CK, which was most significant at the upper section (*p* < 0.05, Figures 4, 5A–C). The ZS networks were less robust than CK except for the center section, indicated by the lower modularity (*p* < 0.05, Figure 5D). This was probably due to the higher betweenness centrality (Figure 5E), which represented a more indirect relationship among the OTUs. Despite not significant, the ratio of facility relationship in both CK and ZS samples increased from lower to upper section (Figure 5F). Leaf section showed a significant effect on the network complex only in CK treatments, with lower node and edge numbers at upper than center section (Figures 5A,B, *p* < 0.05). Similar results were found for the betweenness centrality (Figure 5E). However, the average degree of the “cherry-red” fungal endophytic network increased as the leaf section lifted (*p* < 0.05), while no significant difference was observed for CK samples (Figure 5D).

### Community Structuring Mechanism

**FIGURE 6:**
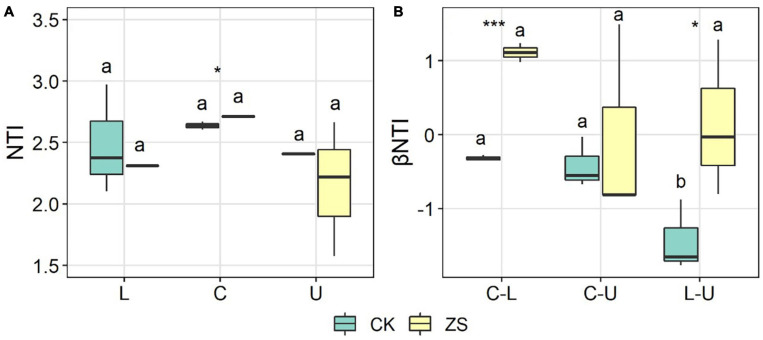
Nearest taxon index (NTI) **(A)** and β nearest taxon index (βNTI) **(B)** of endophytic fungal community in the lower (L), center (C), and upper (U) leaves of ordinary (CK) and “cherry-red” (ZS) tobacco. Asterisks indicate significant differences between CK and ZS, while different lettters above the boxes indicatesignificant differences between plant sections. *p < 0.05; ***p < 0.001.

To reveal the local structuring mechanism of the fungal community in the ordinary and “cherry-red” tobacco leaves, the nearest taxon index (NTI) in each sample was calculated. The NTI values in our study ranged from 1.58–2.97, where most were higher than 2 (Figure 6A), and this suggested that the determinism prevailed over stochasticity in the local community assembly (Stegen et al., 2012). The plant section showed no significant effect on the NTI of both CK and ZS samples, whereas at the center section, the NTI was higher in ZS than CK, indicating a stronger clustering strength in the “cherry-red” tobacco in comparison to the ordinary type (*p* < 0.05, Figure 6A). The βNTI was also calculated to address the mechanism of community structuration among leaves from different sections in individual plants. Higher βNTI of center *vs* lower and lower *vs* upper sections were found in ZS than CK; however, all the values ranged between −2 and 2 (Figure 6B), suggesting that stochasticity controlled the fungal community structuration among different plant sections (Stegen et al., 2012).

## Discussion

As endophytic microorganisms acquire any resource from the host, their community is strongly controlled by the nutrition of plant, for example, the concentration of soluble sugars, proteins, amino acids, organic acids, and so on (Huang et al., 2018; Rho et al., 2020). Despite there being no integral profile of their biomass compounds, previous studies revealed significantly different secondary metabolites between ordinary and “cherry-red” tobacco leaves (Liu et al., 2020), and this most likely leads to a distinguished endophytic fungal community (Figure 2D). Demethylation of nicotine to nornicotine relies on an enzyme catalysis series (An et al., 2007), producing formaldehyde and other by-products (Mesnard et al., 2002). As a result, more kinds of metabolites were detected in “cherry-red” than ordinary leaves (Liu et al., 2020). This may contribute to the higher fungal diversity in ZS than CK. Moreover, the aromas in “cherry-red” leaves are mainly phytols, which are more readily available for microorganisms than alkaloids, the dominant aroma the ordinary tobacco. The degradable carbon resource may also favor more diverse endophytes (Rodriguez et al., 2009). The OTU number of leaf endophytic fungi increased from the lower to upper plant section (Figure 2A). Similarly, the most abundant red dapples were found in the upper leaves, followed by the center, and the least in the lower leaves (Supplementary Figure 2). The results suggested a potential relationship between the emergence of “cherry-red” characteristics and fungal richness. We found a higher Shannon index in “cherry-red” leaves than the ordinary ones, though this was only significant at the center section (Figure 2C). Consistently, center leaves generally showed the highest quality after being cured (Zhao et al., 2006). The result further supported that the diversity of foliar endophytic fungi is related to the formation and quality of “cherry-red” tobacco.

The endophytic fungi in both ordinary and “cherry-red” tobaccos were all non-clavicipitaceous (NC-endophytes), which can be detected in the tissue of almost all terrestrial plants (Arnold and Lutzoni, 2007). It has been reported that *Ascomycota* are the primary NC-endophytes (Rodriguez et al., 2009). Similarly, this phylum dominated all samples of this study (Figure 2E). Another high abundant fungal phylum of our samples was *Basidiomycota*. It should be noticed that both the two phyla belong to the Class 2 subgroup of fungal endophytes, which are distinct in their capacity of vertical transmission (Rodriguez et al., 2009). This study investigated only one growth season, however, the dominant of hereditable taxa suggested a potential of intergenerational stability of tobacco endophytes. The Class 2 endophytic fungi confer to plants the adaption to selective pressures such as salinity, pH, and temperature (Rodriguez et al., 2008). Very limited information on the tobacco endophytic community has been available until now. The primer used in this study (ITS1-F/ITS2) showed no amplification bias on Ascomycota or Basidiomycota (Li et al., 2020). If further studies confirm our above inference, the fungal endophytes should be concluded in the breeding of tobacco.

Nicotine degradation microorganisms (NDMs) have been increasingly noticed over the last half a century, including both bacteria and fungi (Sindelar et al., 1979; Uchida et al., 1983; Liu et al., 2015). Despite fungi receiving less attention than bacteria in the studies of nicotine degradation, almost all the fungal NDMs separated from tobacco leaf are proven to produce nornicotine as only or a kind of the metabolites, while only a few bacterial NDMs are capable to produce nornicotine (Liu et al., 2015). In this study, we found more enriched fungi in the “cherry-red” than ordinary tobacco leaves (Figure 3). Among the fungal NDMs, the nicotine degradation of *Aspergillus oryzae* 112822 isolated from tobacco leaf can reach the maximum amount of 2.9 g/L in 40 h (Meng et al., 2010). This process produces nornicotine as an important intermediate, and production was significantly higher than with other fungal NDMs (Uchida et al., 1983; Liu et al., 2015). Correspondingly, our results recognized *Aspergillus* as a unique biomarker in the upper leaf of “cherry-red” tobacco (Figure 3), where most red dapples were observed after curing (Supplementary Figure 1). Sordariomycetes biomarkers were common in all “cherry-red” leaves, but none were enriched in ordinary samples. Especially, *Hypocreales* were shared by the upper and center leaves of “cherry-red” tobacco (Figure 3). We suppose that these taxa may stimulate the “cherry-red” produce. However, there is still no evidence to support this idea.

Microbial interaction is now accepted as essential for microbial ecology and functions. However, directly detecting the interactions among microorganisms in a complex natural community is still impossibly constrained by the current technologies. Previous studies suggested that the co-occurrence network can model the complex interactions and represent types and properties of interaction in the microbial community by topological features (Barberan et al., 2012; Layeghifard et al., 2017). Here, the significantly higher average degree of ZS samples than CK from the upper and center plant sections indicated higher network complexity of the fungal community in “cherry-red” leaves (Dai et al., 2020). The network structure is found to primarily be shaped by the food supply (Foster et al., 2012). For example, Shi et al. (2016) found a more complex microbial network in the oat rhizosphere than in bulk soil, which is likely due to the higher C availability. As previously illuminated, second metabolites in “cherry-red” tobacco leaves are more decomposable than in ordinary tobacco due to the high phytol content (Liu et al., 2020). This may explain the higher complexity of “cherry-red” endophytic fungi. Nicotine is mainly produced in the root and transported to the aboveground parts, while its demethylation is believed to primarily occur in leaves (Zuo, 1993). The metabolism of the microbial community is governed by the interactions among its members (Nemergut et al., 2014; Dai et al., 2020). The activity of nicotine demethylation in tobacco leaf and thus the emerging of red dapples are vulnerable to agronomic and environmental conditions (An et al., 2007). Dramatically, a less robust network of the fungal community was detected in “cherry-red” than in ordinary tobacco. It might be too far to claim a relationship between the stability of the “cherry-red” characteristic and the network robustness based on our study, but the phenomenon is interesting.

We finally looked into the structuring mechanism shaping the assembly of endophytic fungi in “cherry-red” and ordinary tobacco leaves using the NTI and βNTI index. For a community setting, an NTI significantly higher than 0 indicated the phylogenetic clustering rather than overdispersion responsible from the community assembly on average (Kembel, 2009), which met the results of our study (Figure 6A). The NTI > 2 in almost all samples further confirmed the dominance of deterministic processes in local community structuration of tobacco endophytic fungi. We found higher NTI in “cherry-red” than ordinary tobacco in the center leaves (Figure 6A). The results suggested a great selection of the foliar endophytic fungi of “cherry-red” tobacco (Stegen et al., 2012). However, the turnover of fungi among different sections in individual tobacco plants appeared to be stochastic, indicated by the −2 < βNTI < 2 (Figure 6B; Stegen et al., 2012; Dini-Andreote et al., 2015). Similar results were also observed for other plants, such as woody trees (Haruna et al., 2018). Therefore, we suggest that tobacco leaves randomly achieve fungi from the seed pool (soil, air, etc.), and then filter them *in vivo* at individual plant sections to form a certain microbiota.

## Data Availability Statement

The datasets presented in this study can be found in online repositories. The names of the repository/repositories and accession number(s) can be found below: https://www.ncbi.nlm.nih.gov/, PRJNA698403.

## Author Contributions

YJ and XC extracted DNA, performed PCR, analyzed the data, and wrote the manuscript. CZ designed the experiment and revised the manuscript. HG revised the manuscript. GZ, JL, YX, and YL managed the field and sampling.

## Conflict of Interest

JL and YX were employed by the company Yunnan Tobacco Company. The remaining authors declare that the research was conducted in the absence of any commercial or financial relationships that could be construed as a potential conflict of interest.

## Footnotes

1 https://www.r-project.org/

2 https://gephi.org/

3 http://huttenhower.sph.harvard.edu/galaxy
