# Supplementary material for: Endophytic Fungal Community of Tobacco Leaves and Their Potential Role in the Formation of “Cherry-Red” Tobacco
Source: Front Microbiol. 2021 Jul 16;12:658116. doi: 10.3389/fmicb.2021.658116 (PMC8323715; doi:10.3389/fmicb.2021.658116)

**Fig. S1** Cured leaves Ordinary (CK) and “cherry-red” (ZS) tobacco from upper, center and lower plant sections.


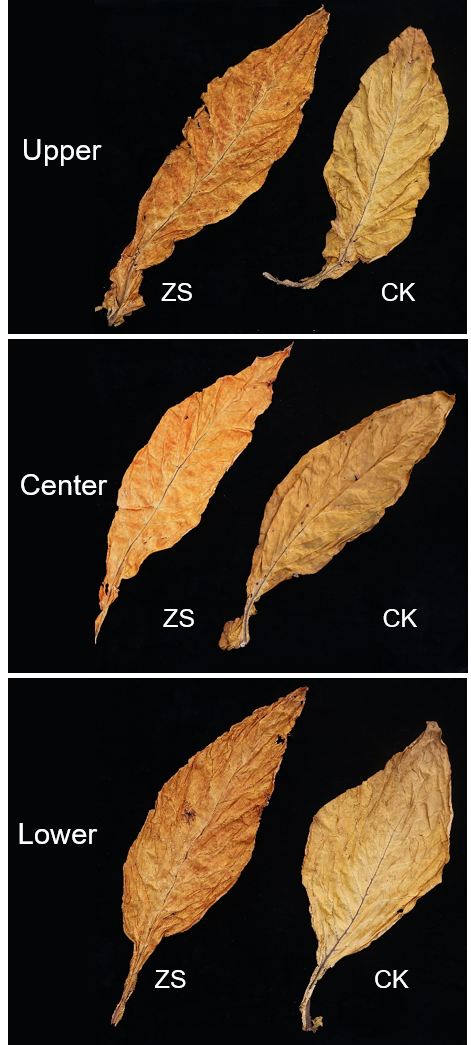


**Fig. S2** Cladogram showing the LAD analysis results of the endophytic fungal community in lower (a), center (b) and upper (c) leaves in comparison of ordinary (CK) and “cherry-red” tobacco.


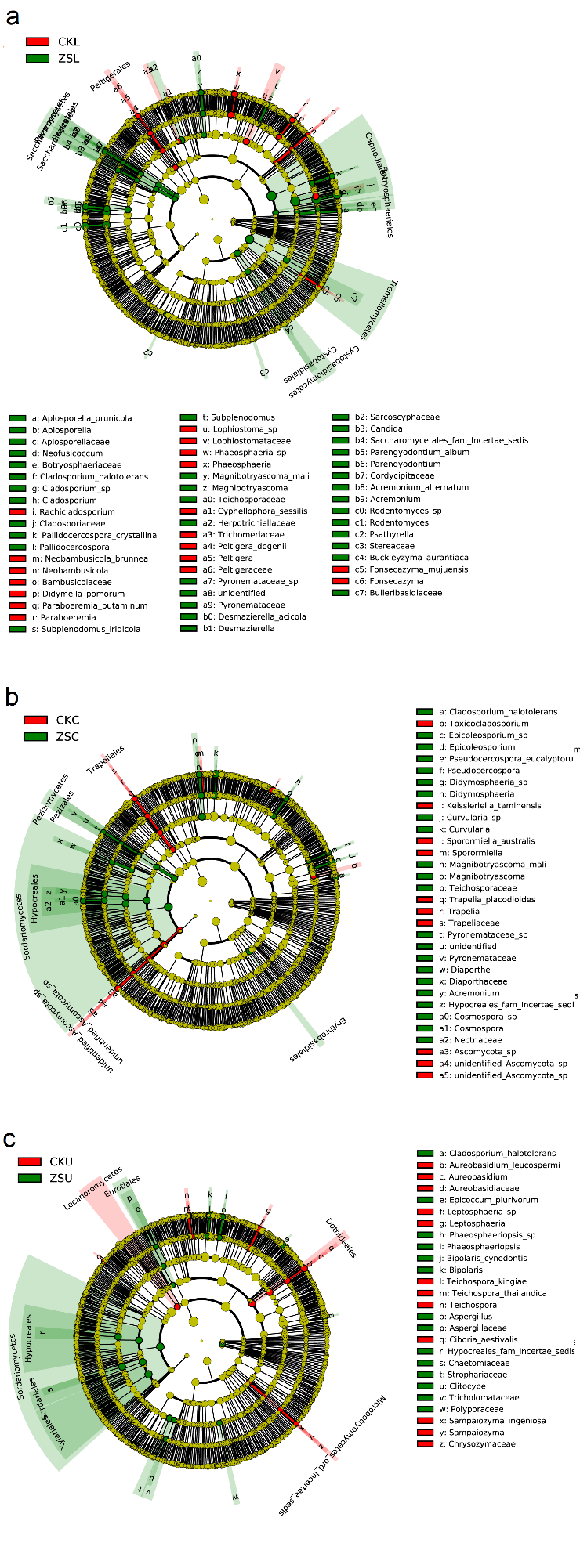

Supplement: Supplementary file 1 [file Table_1.DOCX]
